# Two Faces of Catechol-O-Methyltransferase Inhibitor on One-Carbon Metabolism in Parkinson’s Disease: A Meta-Analysis

**DOI:** 10.3390/nu15040901

**Published:** 2023-02-10

**Authors:** Jin Hee Kim, Shaoyue Jin, Hyeyoon Eo, Myung Sook Oh, Yunsook Lim

**Affiliations:** 1 Department of Biomedical and Pharmaceutical Science, Kyung Hee University, Seoul 02447, Republic of Korea; 2 School of Medicine, Ningbo University, Ningbo 315211, China; 3 Department of Oriental Pharmaceutical Science and Kyung Hee East–West Pharmaceutical Research Institute, College of Pharmacy, Kyung Hee University, Seoul 02447, Republic of Korea; 4 Department of Food and Nutrition, Graduate School, Kyung Hee University, Seoul 02447, Republic of Korea

**Keywords:** meta-analysis, Parkinson’s disease, one-carbon metabolism, levodopa, homocysteine, vitamin B, folate

## Abstract

Levodopa (L-dopa) and catechol-O-methyltransferase (COMT) inhibition are widely used therapeutics in Parkinson’s disease (PD). Despite their therapeutic effects, it was raised that nutrients involved in one-carbon metabolism can be deteriorated by PD therapies. The aim of this meta-analysis was to investigate the impact of L-dopa and COMT inhibitors on levels of homocysteine (Hcy), vitamin B_12_ and folate in patients with PD. A total of 35 case-control studies from 14 different countries were selected through PubMed, MEDLINE and Google Scholar and were meta-analyzed. In the L-dopa group, the Hcy level was higher compared to the PD without L-dopa group (SMD: 5.11 μmol/L, 95% CI: 3.56 to 6.66). Moreover, vitamin B_12_ and folate levels in the L-dopa group were lower compared to the healthy control (SMD: −62.67 pg/mL, 95% CI: −86.53 to −38.81; SMD: −0.89 ng/mL, 95% CI: −1.44 to −0.33, respectively). The COMT inhibitor group showed lower levels of Hcy (SMD: −3.78 μmol/L, 95% CI: −5.27 to −2.29) and vitamin B_12_ (SMD: −51.01 pg/mL, 95% CI: −91.45 to −10.57), but higher folate levels (SMD: 1.78 ng/mL, 95% CI: −0.59 to 4.15) compared to the L-dopa group. COMT inhibitors may ameliorate L-dopa-induced hyper-homocysteine and folate deficiency but exacerbate vitamin B_12_ deficiency.

## 1. Introduction

Parkinson’s disease (PD) is a typical neurodegenerative disease characterized by motor dysfunction, such as tremors, rigidity and slow movements, resulting from the loss of dopaminergic neurons [1]. It is mainly caused by environmental factors. Its incidence is approximately 1% among people over the age of 60 worldwide and steadily increasing [2].

For patients with PD, dopamine replacement is the treatment of choice, and the most commonly used drug is levodopa (L-dopa), a dopamine precursor [1]. Because dopamine itself cannot cross the blood-brain barrier (BBB) owing to its large molecular weight, L-dopa is administered [3]. However, L-dopa can easily convert to other structures, such as 3-O-methyldopa catalyzed by the enzyme catechol-O-methyl transferase (COMT) before it crosses the BBB or reaches the brain. [4]. To prevent this undesirable conversion, L-dopa is often prescribed along with COMT inhibitors, such as entacapone [5]. Moreover, it can cause serious side effects, such as dyskinesia [6]. It accelerates PD progression by inducing neuronal cell death through self-oxidation [7].

Furthermore, L-dopa can exacerbate elevated homocysteine (Hcy) levels in patients with PD [8]. Hcy is a thiol-containing amino acid and an intermediate product in the folate-methionine cycle [9]. Under healthy conditions, Hcy reverts to methionine in the presence of B vitamins, such as folate and vitamin B_12_ [10]. When folate enters the folate cycle in the form of tetrahydrofolate (THF), it converts into 5-methyl-THF. Together with vitamin B_12_, 5-methyl-THF can provide its methyl group to Hcy, catalyzed by methionine synthase, to produce methionine and, consequently, maintain a lower Hcy level [10]. However, Hcy conversion is not appropriately facilitated in PD, resulting in elevated circulating Hcy levels, which leads to cellular damage through oxidative stress and inflammation [11]. L-dopa aggravates Hcy metabolism by directly involving the folate-methionine cycle or one-carbon metabolism [12]. Briefly, in the presence of COMT, S-adenosylmethionine provides its methyl group to L-dopa to produce S-adenosylhomocysteine (SAH) [13]. SAH is rapidly hydrolyzed to Hcy, which is elevated in patients with PD [14]. Therefore, the side effects of L-dopa in patients with PD might be closely related to the dysregulation of Hcy, vitamin B_12_ and folate.

To optimize nutritional approaches for patients with PD, a comprehensive meta-analysis of various studies worldwide should be performed comparing the circulating levels of Hcy, vitamin B_12_ and folate in patients with PD taking L-dopa. The aim of the present study was to assess the impact of L-dopa administration on the folate-methionine cycle based on circulating levels of Hcy, vitamin B_12_ and folate. The effect of the COMT inhibitor on L-dopa-induced dysregulation of the folate-methionine cycle was also investigated, as COMT inhibitors are often combined with the L-dopa treatment.

## 2. Materials and Methods

### 2.1. Literature Search

A literature search for the current meta-analysis was performed using PubMed, MEDLINE and Google Scholar for articles published until 25 August 2022. The literature was searched using the following keywords: “Parkinson’s disease (PD),” “Levodopa,” “L-dopa,” “Homocysteine (Hcy),” “Vitamin B_12_,” “Folate” and “COMT inhibitor.” In addition, Chinese literature was searched using the China Knowledge Resource-Integrated Database. In addition, this study was conducted based on the PRISMA 2020 Checklist.

### 2.2. Inclusion and Exclusion Criteria

Inclusion criteria were: (1) full-text articles of randomized or non-randomized clinical trials involving humans; (2) studies on the relationship between L-dopa use and Hcy, vitamin B_12_ or folate in patients with PD; (3) studies comparing L-dopa with a healthy control or COMT inhibitor group; (4) articles containing data expressed as mean ± standard deviation and complete information. Exclusion criteria were: (1) reviews or articles without data, (2) studies involving patients on vitamin B supplementation and (3) duplicate publications. Study selection was conducted according to the Preferred Reporting Items for Systematic Reviews and Meta-Analyses flow diagram.

### 2.3. Data Extraction

Data were tabulated in a spreadsheet under the following headings: first author, publication year, title and parameters analyzed. “Healthy control” was defined as a group with participants without PD. “Without L-dopa” was defined as a group of patients with L-dopa-naïve PD. “L-dopa” was defined as a group of patients with PD taking only L-dopa. “COMT inhibitor” was defined as a group of patients with PD taking both L-dopa and the COMT inhibitor together. Data are available within the selected studies and/or their Supplementary Materials as well as upon request from the authors.

### 2.4. Statistical Analyses

Statistical analyses were performed using Review Manager (RevMan) 5.2. The degree of inconsistency within studies, or heterogeneity (*I*^2^), was interpreted based on the following reference points and their *p*-values: 0%, 25%, 50% and 75%, representing no, low, moderate and high heterogeneity, respectively [15]. When heterogeneity was low, a random-effects model was used; otherwise, a fixed-effects model was used. The standardized mean difference (SMD) was used as the effect size for Hcy, vitamin B_12_ and folate comparisons. Statistical significance was set at a *p*-value < 0.05. Each analysis was presented as a forest plot. The green squares represent the weighted mean difference in each study, and the black diamonds represent the summary of the weighted mean difference in the forest plots. Funnel plots were constructed using RevMan to assess the publication bias. When the plot showed symmetrical and equal scattering, the results indicated no publication bias.

## 3. Results

### 3.1. Search Results

**Figure 1:**
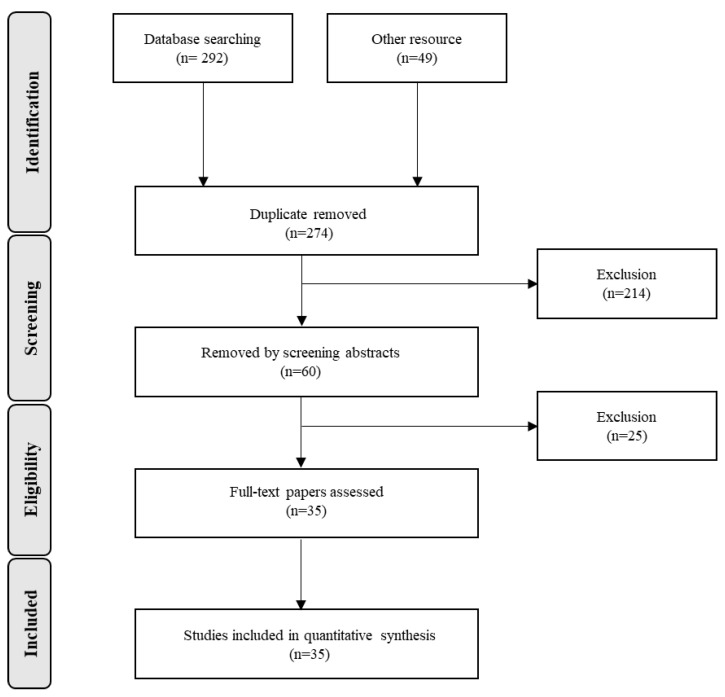
Flow diagram of study selection process.

In the literature search, 341 clinical trials were initially screened, and duplicate articles were removed. After applying the exclusion criteria, a total of 35 clinical trials were included in the meta-analysis (Figure 1), with blood/serum Hcy, vitamin B_12_ and folate levels as measured biomarkers in the selected studies. Figure 1 shows the details of the selection procedure.

### 3.2. Study Characteristics in the Meta-Analysis

Table 1 summarizes the characteristics of the included clinical trials. Among the 35 clinical trials, 12 were carried out in China and Taiwan, while 7 were in Italy, 4 in Germany, 2 in Greece and 1 each in Australia, Poland, the Republic of Korea, Japan, Thailand, Czechia, Turkey, the United States, Slovakia and Canada. The number of participants enrolled in the selected studies ranged from 12 to 212, and participants were generally over the age of 60 years. The L-dopa dose was ≥300 mg/day in the L-dopa group. Treatment duration ranged from 3.0 to 12.4 years for L-dopa and 4.3 to 13.5 years for the COMT inhibitor.

### 3.3. Comparison of Hcy Levels in Blood

**Figure 2:**
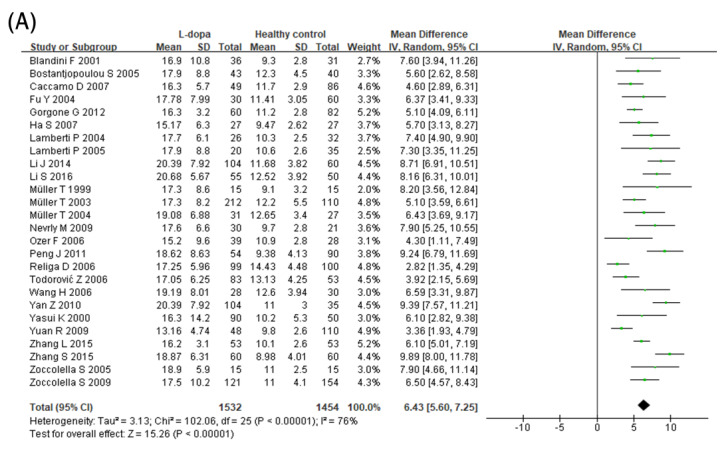

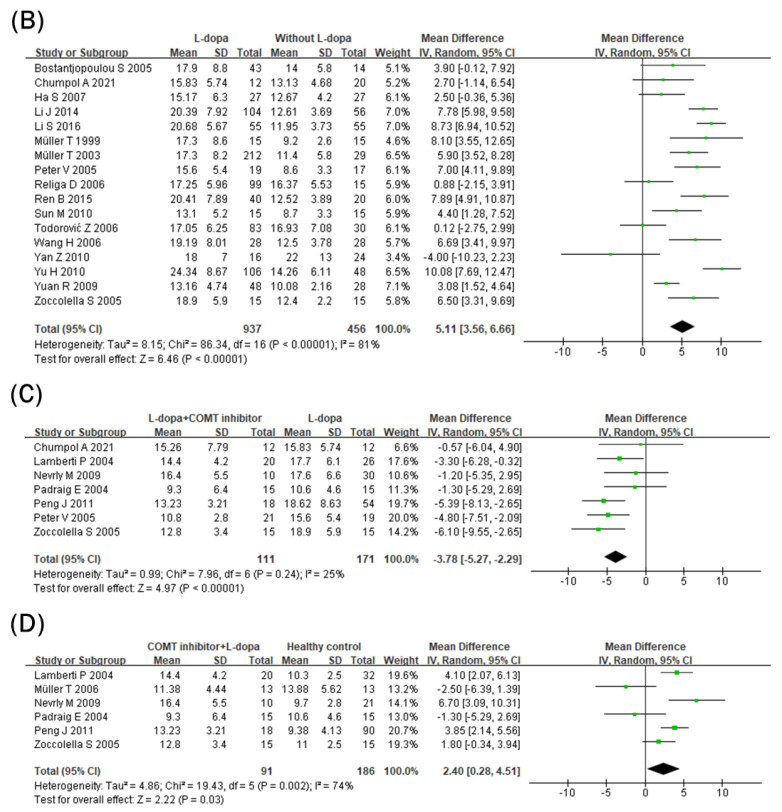
Forest plot of the blood Hcy levels comparing (**A**) L-dopa group and healthy controls, (**B**) L-dopa group and without L-dopa group, (**C**) COMT inhibitor group and L-dopa group and (**D**) COMT inhibitor group and healthy control.

The effect of the L-dopa treatment on blood Hcy levels was examined in 26 studies (Figure 2A). Blood Hcy levels were significantly higher in the L-dopa group than in the healthy control group (Figure 2). The overall SMD of the 26 studies was 6.43 μmol/L (95% confidence interval [CI]: 5.60 to 7.25; *p* < 0.00001) in random-effects models with high significant heterogeneity (*I^2^* = 76%, *p* < 0.000001). Subsequently, the impact of the L-dopa treatment on blood Hcy levels in patients with PD was examined (Figure 2B). Hcy levels were significantly higher in the L-dopa group than in the without L-dopa group. The overall SMD of the 17 studies was 5.11 μmol/L (95% CI: 3.56 to 6.66; *p* < 0.00001) in random-effects models with high significant heterogeneity (*I^2^* = 81%, *p* < 0.00001). Moreover, the influence of the COMT inhibitor on high blood Hcy levels induced by the L-dopa treatment in patients with PD was investigated (Figure 2C). The group treated with both L-dopa and the COMT inhibitor had lower levels of Hcy in the blood compared to the group taking L-dopa alone. The overall SMD of the seven studies was −3.78 μmol/L (95% CI: −5.27, −2.29; *p* < 0.00001) in the random-effects models with no significant heterogeneity (*I^2^ =* 25%, *p* = 0.24). Finally, the blood level of Hcy in the L-dopa plus COMT inhibitor treatment group was compared with that in the healthy control group (Figure 2D). Hcy levels were significantly higher in the COMT inhibitor group than in the healthy control group. The overall SMD of the six studies was 2.40 μmol/L (95% CI: 0.28 to 4.51; *p* = 0.03) in random-effects models with modest substantial heterogeneity (*I^2^* = 74%, *p* = 0.002).

### 3.4. Comparison of Vitamin B_12_ Levels in Blood

**Figure 3:**
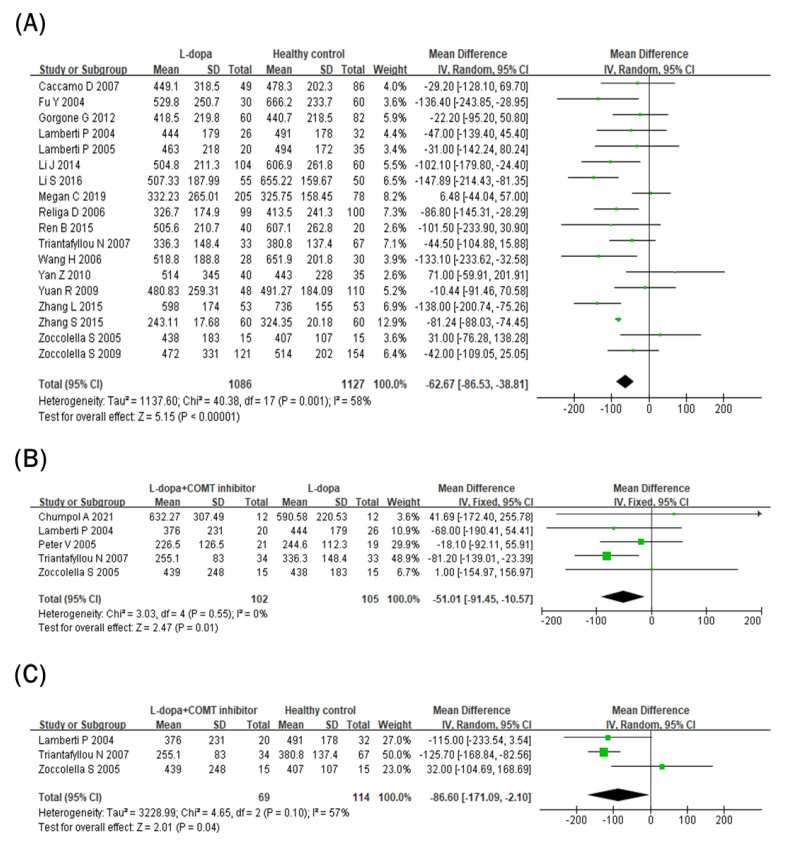
Forest plot of the blood vitamin B_12_ levels comparing (**A**) L-dopa group and healthy control, (**B**) COMT inhibitor group and L-dopa group and (**C**) COMT inhibitor group and healthy control.

Blood vitamin B_12_ levels were lower in the L-dopa group than in the healthy control group (Figure 3A). The overall SMD of the 18 studies was −62.67 pg/mL (95% CI: −86.53 to −38.81; *p* < 0.00001) in random-effects models with modest and significant heterogeneity (*I^2^* = 58%, *p* = 0.001). In addition, blood vitamin B_12_ levels were even lower in the COMT inhibitor group (Figure 3B). The overall SMD of the five studies was −51.01 pg/mL (95% CI: −91.45 to −10.57; *p* = 0.01) in fixed-effects models with no heterogeneity (*I^2^ =* 0%, *p* = 0.55). Moreover, blood vitamin B_12_ levels were still lower in the COMT inhibitor group than in the healthy control group (Figure 3C). The overall SMD of the three studies was −86.60 pg/mL (95% CI: −171.09 to −2.10; *p* = 0.04) in random-effects models with no significant heterogeneity (*I^2^ =* 57%, *p* = 0.10) and larger than the overall SMD of the 18 studies comparing the L-dopa and healthy control groups (−62.67 pg/mL).

### 3.5. Comparison of Folate Levels in Blood

**Figure 4:**
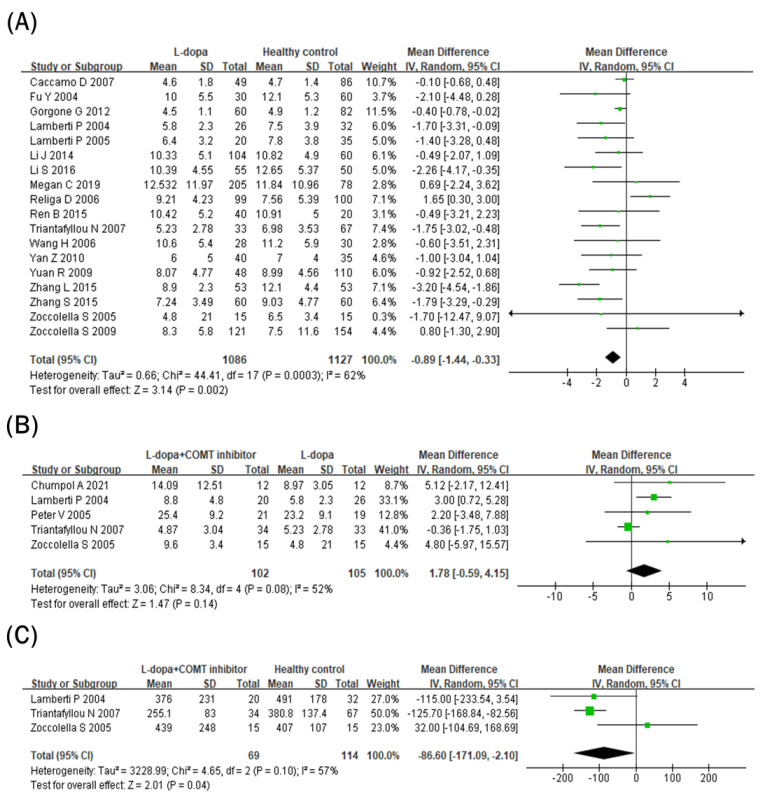
Forest plot of the blood folate levels comparing (**A**) L-dopa group and healthy control, (**B**) COMT inhibitor group and L-dopa group and (**C**) COMT inhibitor group and healthy control.

Blood folate levels were compared between the L-dopa and healthy control groups (Figure 4A). Blood folate levels were significantly lower in the L-dopa group than in the healthy control group. The overall SMD of the 18 studies was −0.89 ng/mL (95% CI: −1.44 to −0.33; *p* = 0.002) in random-effects models with modest heterogeneity (*I^2^* = 62%, *p* = 0.00003). Subsequently, the impact of the COMT inhibitor on circulating folate levels in the L-dopa group was examined (Figure 4B). Blood folate levels did not differ significantly between the COMT inhibitor and L-dopa groups (SMD = 1.78 ng/mL, 95% CI: −0.59 to 4.15; *p* = 0.14) in random-effects models with no heterogeneity (*I*^2^ = 52%, *p* = 0.08). Furthermore, blood folate levels were compared between the COMT inhibitor and healthy control groups (Figure 4C). Blood folate levels did not differ significantly between the COMT inhibitor and healthy control groups (SMD = 1.20 ng/mL, 95% CI: −0.78, 3.18; *p* = 0.24) in random-effects models with no heterogeneity (*I^2^* = 65%, *p* = 0.06).

### 3.6. Publication Bias

Publication bias was assessed using funnel plots (Supplementary Figures S2–S4). There was no evidence of publication bias with funnel plots of circulating Hcy, vitamin B_12_ or folate for the healthy control or COMT inhibitor group.

## 4. Discussion

In the current meta-analysis, the impact of L-dopa and the COMT inhibitor on the folate–methionine cycle in PD was evaluated based on the blood levels of Hcy, vitamin B_12_ and folate. Two previous meta-analyses investigated if there is any correlation between the ratio of Hcy/vitamin B_12_/folate and PD incidence [48,49]. However, one of them was confined to studies in China and did not consider the impact of L-dopa or COMT inhibitor therapy [48]. The other included a small number of studies and did not evaluate vitamin B_12_ or folate levels, which should be considered in terms of the folate–methionine cycle [49]. To the best of our knowledge, the current study would be the first report on the effects of L-dopa and its concomitant COMT inhibitor on the folate–methionine cycle based on the blood levels of Hcy, vitamin B_12_ and folate and integrating numerous reports worldwide.

Hcy is closely associated with the promotion of PD pathologies, and its elevation should be considered as a main risk factor for PD [50]. In this meta-analysis, the circulating Hcy concentrations in various subgroups of patients with PD were investigated (Figure 2). The blood Hcy level was elevated in the L-dopa group compared to both the healthy control and without L-dopa groups. These results are similar to those of previous meta-analyses, showing that the same results were obtained even when the number of studies increased [48]. In addition, the normal range of blood Hcy levels is 5–15 μmol/L, and circulating Hcy concentrations ≥15 μmol/L are considered to indicate hyper-homocysteinemia [10]. In this study, 25 of 26 included studies showed that the mean blood Hcy concentration in the L-dopa group was >15 μmol/L (Figure 2A), while the mean blood Hcy levels in the without L-dopa group in 15 of 17 included studies were below the borderline (Figure 2B). High Hcy causes various pathogenic outcomes, such as oxidative stress and inflammation which can accelerate neuronal cell death [10]. In addition, many studies reported that Hcy has a high correlation with the cardiovascular system by causing pathological problems in cardiovascular endothelium and smooth muscle cells [51,52,53]. For these reasons, the current results imply that various side effects reported in PD patients with prolonged L-dopa intervention might be related to the elevation of circulating Hcy and its pathogenic responses. In contrast, the COMT inhibitor group showed significantly lower Hcy levels compared to the L-dopa group (Figure 2C). This might result from the fact that the COMT inhibitor suppressed the overproduction of SAH by COMT after taking L-dopa in one-carbon metabolism [12]. Therefore, the current study suggests that COMT inhibitors can not only involve in the secure delivery of L-dopa but reduce potential side effects such as hyperhomocysteinemia caused by L-dopa intervention. 

In addition to Hcy, vitamin B_12_ or folate can be found as methyl donors in one-carbon metabolism or the folate–methionine cycle [14]. Thus, deficiencies in folate and vitamin B_12_ can interfere with DNA synthesis or replication, subsequently contributing to cell death or the development of diseases such as cancer [54,55,56]. In a previous study, folic acid deficiency with transformed Hcy inhibited DNA repair in the hippocampal neurons [57]. Similarly, low serum vitamin B_12_ concentrations are related to brain damage [58]. Thus, folate and vitamin B_12_ are important nutrients facilitating the proper folate–methionine cycle and inhibiting neuronal damage. In this meta-analysis, both blood vitamin B_12_ and folate levels were significantly reduced in the L-dopa group compared to the healthy control group. In particular, circulating vitamin B_12_ levels were further reduced in the COMT inhibitor group compared to the L-dopa group (Figure 3B), but blood folate concentrations did not change in the COMT inhibitor group and rather showed an increasing tendency (Figure 4B). A previous meta-analysis showed that patients with PD had lower circulating vitamin B_12_ and folate levels than healthy controls [48]. Therefore, the current data imply that L-dopa and/or COMT inhibitor therapy might deteriorate vitamin B imbalance in patients with PD.

To understand the dual effect of the COMT inhibitor on the folate–methionine cycle, the normal range of each factor examined in this study should be reviewed. The normal range of blood concentration is 5–15 μmol/L for Hcy [10], 200–900 pg/mL for vitamin B_12_ [59] and 3.0 ng/mL or greater for folate (sufficient concentration of folate controversial) [60]. Accordingly, the levels of vitamin B_12_ and folate were within the normal range, even in the COMT inhibitor group. However, in the case of Hcy, the risk of hyperhomocysteinemia exceeded the borderline of 15 μmol/L in the L-dopa group. Thus, the current data suggest that the COMT inhibitor reduces the side effects of L-dopa related to hyperhomocysteinemia.

In addition to the normal range of each factor, the organs involved in the folate–methionine cycle should be considered. The main organ involved in one-carbon metabolism is the liver Vitamin B_12_ is mostly stored in the liver until the body requires it. Half of the folate concentration is stored in the liver, and the rest is stored in blood and tissues. Therefore, alterations in the blood folate concentration might not indicate metabolic processes, unlike those in vitamin B_12_ levels. Therefore, alterations in circulating vitamin B_12_ and folate showed different patterns after the drug treatment.

Finally, the disease duration of each group should be considered. In this analysis, the COMT inhibitor group had a longer disease duration than the L-dopa group (Supplementary Figure S1). This may be because, in general, the longer the disease duration, the greater the chance of taking a combination of L-dopa and the COMT inhibitor. In addition, patients with a longer disease duration might undergo further PD progression, which may affect the status of vitamin B_12_ and folate, as they play roles in the folate–methionine cycle, erythropoiesis and iron homeostasis [61]. Therefore, the difference in disease duration would also affect the results showing the dual nature of the COMT inhibitor. Nevertheless, the current data provide evidence that L-dopa or COMT administration can affect vitamin B_12_, folate and Hcy levels to a certain degree.

Collectively, this study demonstrated hyperhomocysteinemia and lower blood levels of vitamin B_12_ and folate in PD patients with L-dopa administration. An increase in blood Hcy levels induces neuronal cell death, and vitamin B_12_ and folate deficiencies suppress their neuroprotective effects [11]. These results are in line with previous reports showing that L-dopa can damage dopaminergic neurons, thereby accelerating PD progression [7]. Thus, possible causes of disease exacerbation from L-dopa therapy would be an elevation in Hcy but a reduction in vitamin B_12_ and folate. Moreover, this meta-analysis elucidated that COMT inhibitors might be beneficial in ameliorating hyperhomocysteinemia and folate deficiency induced by the L-dopa treatment. However, COMT inhibitors can exacerbate vitamin B_12_ deficiency in patients with PD on L-dopa. In conclusion, the current data suggest that a COMT inhibitor with vitamin B supplementation could reduce L-dopa-induced PD deterioration.

The present study had the strengths of integrating global data and considering concomitant L-dopa therapy. However, this study had some limitations. First, the clinical trials included both randomized and non-randomized ones, such as cross-sectional studies. Second, no quality assessment was performed. Third, the studies showed heterogeneity (Figure 2A,B,D, Figure 3A and Figure 4A). The observed heterogeneity disappeared when studies were stratified by the geographic region, as previously described [62]. The North American region showed a markedly higher incidence compared to other regions, whereas some countries, such as Italy, showed decreasing trends in the estimated annual percentage change in PD from 1990 to 2019 [63]. Fourth, the group taking vitamin B supplements was not considered in the current study. Most of the studies excluded participants taking vitamin B supplements, which may strengthen or contradict the main conclusion of the current meta-analysis. Lastly, as the analysis of vitamin B_12_ or folate was not conducted as much as that of Hcy, the difference between the without L-dopa and L-dopa groups was not determined, and the sample sizes of other studies on vitamin B_12_ and folate were small as well. Therefore, more clinical studies are required to clarify the impact of L-dopa and the COMT inhibitor on the folate–methionine cycle based on various factors, such as geographical regions and biodiversity.

## Figures and Tables

**Table 1:** Characteristics of included clinical trials in the current meta-analysis. When the reports do not indicate standard deviation (SD), only mean values were written (mean ± SD). L-dopa: levodopa; COMT: catechol-O-methyltransferase; NA: not assessed; -: undescribed.

Author Year ^ref^	Country	L-Dopa				Without L-Dopa			COMT Inhibitor				Healthy Control	
		L-Dopa Dose (mg/day)	Duration (year)	Age	Case	Duration (yr)	Age	Case	L-Dopa Dose (mg/day)	Duration (year)	Age	Case	Age	Case
Blandini F., 2001 [16]	Italy	768 ± 216	12.4 ± 5.4	62.7 ± 13.8	36	NA	NA	NA	NA	NA	NA	NA	58.9 ± 7.2	31
Bostantjopoulou S., 2005 [8]	Greece	563.9 ± 232.2	8.1 ± 5.1	58.0 ± 10.3	43	-	-	14	NA	NA	NA	NA	-	40
Caccamo D., 2007 [17]	Italy	509.4 ± 312.1	5.8 ± 4.1	64.2 ± 7.5	49	NA	NA	NA	NA	NA	NA	NA	64.1 ± 7.1	86
Chumpol A., 2021 [14]	Thailand	300	4.5	68.67 ± 6.04	12	1	75.40 ± 8.65	20	375	5.29	69.17 ± 10.88	12	n.a.	n.a
Fu Y., 2004 [18]	China	-	-	62.4 ± 8.8	30	NA	NA	NA	NA	NA	NA	NA	62.7 ± 9.8	60
Gorgone G., 2012 [19]	Italy	452.0 ± 170.0	5.0 ± 3.0	64.4 ± 7.7	60	NA	NA	NA	NA	NA	NA	NA	64.7 ± 7.2	82
Ha S., 2007 [20]	Republic of Korea	628	3.3	67	60	NA	NA	NA	NA	NA	NA	NA	68	27
Lamberti P., 2004 [21]	Italy	570 ± 250	9.3 ± 4.2	64 ± 8.5	26	NA	NA	NA	780 ± 250	12.8 ± 5.4	63.3 ± 9.6	20	64.5 ± 11.5	32
Lamberti P., 2005 [22]	Italy	640 ± 240	9.4 ± 4.2	65.1 ± 8.5	20	NA	NA	NA	NA	NA	NA	NA	64.1 ± 11	35
Li J., 2014 [23]	China	-	-	-	104	-	-	56	NA	NA	NA	NA	63.8 ± 9.8	60
Li S., 2016 [24]	China	-	6.4 ± 1.3	66.7 ± 4.2	55	6.4 ± 1.3	66.7 ± 4.2	55	NA	NA	NA	NA	65.8 ± 5.1	50
Megan C., 2019 [25]	Australia	888.6 ± 587.9	8.9 ± 5.80	64.0 ± 9.38	205	NA	NA	NA	NA	NA	NA	NA	-	78
Müller T., 1999 [26]	Germany	-	-	-	15	-	-	15	NA	NA	NA	NA	-	15
Müller T., 2003 [27]	Germany	-	-	-	212	-	-	29	NA	NA	NA	NA	-	110
Müller T., 2004 [28]	Germany	473.21 ± 228.11	8.03 ± 6.10	62.65 ± 7.3	31	NA	NA	NA	NA	NA	NA	NA	59.56 ± 10.86	27
Müller T., 2006 [29]	Germany	457.69 ± 272.22	-	65.23 ± 10.26	13	NA	NA	NA	-	-	-	13	-	13
Nevrly M., 2009 [30]	Czechia	560.8 ± 208.6	7.5	4.2	30	NA	NA	NA	NA	NA	NA	NA	51.7 ± 11.1	21
Ozer F., 2006 [31]	Turkey	338.5 ± 222.3	6.4 ± 3.9	67.0 ± 9.3	39	NA	NA	NA	NA	NA	NA	NA	61.9 ± 8.3	28
Padraig E., 2004 [32]	USA	604 ± 204	3.3 ± 1.6	64 ± 12	30	NA	NA	NA	-	4.3	67	15	64 ± 12	30
Peng J., 2011 [33]	China	438 ± 265	-	69.8 ± 7.83	54	NA	NA	NA	NA	NA	NA	NA	64.8 ± 7.58	18
Peter V., 2005 [13]	Slovakia	439.5 ± 203.8	3.62 ± 3.54	71.7 ± 9.2	19	2.18 ± 1.66	62.4 ± 7.1	17	567.9 ± 332.5	7.76 ± 4.91	68.7 ± 9.6	21	NA	NA
Religa D., 2006 [34]	Poland	681.2 ± 328.7	6.06 ± 4.05	70.5 ± 7.57	99	1.97 ± 1.02	66.0 ± 7.11	15	NA	NA	NA	NA	71.2 ± 6.0	100
Ren B., 2015 [35]	China	-	6.0 ± 4.5	63.1 ± 10.2	40	6.0 ± 4.5	63.1 ± 10.2	20	NA	NA	NA	NA	-	20
Sun M., 2010 [36]	China	-	3.0 ± 1.25	62.9 ± 6.1	15	3 ± 1.25	62.9 ± 6.1	15	NA	NA	NA	NA	NA	NA
Todorović Z., 2006 [37]	Canada	531.95 ± 183	3.7 ± 2.7	61.86 ± 9.14	83	1.69 ± 0.59	59.13 ± 8.65	30	NA	NA	NA	NA	60.83 ± 13.13	53
Triantafyllou N., 2007 [38]	Greece	659.6 ± 172.6	7.3 ± 3.4	69.9 ± 5.3	67	NA	NA	NA	-	-	631.9 ± 170.2	34	-	67
Wang H., 2006 [39]	China	-	-	62.4 ± 8.8	28	-	62.4 ± 8.8	28	NA	NA	NA	NA	63.5 ± 8.3	30
Yan Z., 2010 [40]	China	-	-	60.0 ± 11.0	24	-	60.0 ± 11.0	16	NA	NA	NA	NA	58 ± 11	35
Yasui K., 2000 [41]	Japan	412.6 ± 253.6	6.3 ± 5.7	68 ± 9.1	54	NA	NA	NA	NA	NA	NA	NA	71.8 ± 4.9	132
Yu H., 2010 [42]	China	-	6.2 ± 5.1	66.9 ± 10.2	106	6.2 ± 5.1	66.9 ± 10.2	48	NA	NA	NA	NA	NA	NA
Yuan R., 2009 [43]	Taiwan, China	360.21 ± 137.62	6.56 ± 4.3	71.83 ± 10.34	48	2.45 ± 1.37	70.59	28	NA	NA	NA	NA	69.95 ± 8.46	110
Zhang L., 2015 [44]	China	383.4 ± 225.3	4.6 ± 3.4	65 ± 9	53	NA	NA	NA	NA	NA	NA	NA	64 ± 8	53
Zhang S., 2015 [45]	China	-	5.79 ± 2.53	62.97 ± 3.98	60	NA	NA	NA	NA	NA	NA	NA	62.46 ± 4.21	60
Zoccolella S., 2005 [46]	Italy	790 ± 290	9.8 ± 4.8	61.6 ± 8.1	15	4.7 ± 3.6	60.7 ± 8.4	15	-	13.5 ± 5.8	61.3 ± 10.2	15	61 ± 10.4	15
Zoccolella S., 2009 [47]	Italy	-	-	68.7 ± 8.8	121	NA	NA	NA	NA	NA	NA	NA	67.4 ± 8	154

## Data Availability

The data that support the findings of this study are available from the corresponding author upon reasonable request.

## Supplementary Materials

The following supporting information can be downloaded at https://www.mdpi.com/article/10.3390/nu15040901/s1: Figure S1: The difference in mean of disease duration between L-dopa group and COMT inhibitor group; Figure S2: Funnel plot of Hcy-related studies; Figure S3: Funnel plot of Vit B12-related studies; Figure S4: Funnel plot of folate-related studies; Figure S5: PRISMA 2020 Checklist.
